# Mitochondria-Targeted Human Catalase in the Mouse Longevity MCAT Model Mitigates Head-Tilt Bedrest-Induced Neuro-Inflammation in the Hippocampus

**DOI:** 10.3390/life12111838

**Published:** 2022-11-09

**Authors:** Linda Rubinstein, Frederico Kiffer, Stephanie Puukila, Moniece G. Lowe, Brie Goo, Amalia Luthens, Ann-Sofie Schreurs, Samantha M. Torres, Sonette Steczina, Candice G. T. Tahimic, Antiño R. Allen

**Affiliations:** 1 Universities Space Research Association USRA, Columbia, MD 21046, USA; 2 Space Biosciences Division, NASA Ames Research Center, Moffett Field, Mountain View, CA 94035, USA; 3 The Joseph Sagol Neuroscience Center, Sheba Research Hospital, Ramat Gan 52621, Israel; 4 Children’s Hospital of Philadelphia, Philadelphia, PA 19104, USA; 5 Division of Radiation Health Department of Pharmaceutical Sciences, University of Arkansas for Medical Sciences, Little Rock, AR 72205, USA; 6 Blue Marble Space Institute of Science, Seattle, WA 98154, USA; 7 KBR, Houston, TX 77002, USA; 8 Department of Biology, University of North Florida, Jacksonville, FL 32224, USA; 9 Neurobiology and Developmental Sciences, University of Arkansas for Medical Sciences, Little Rock, AR 72205, USA

**Keywords:** microgravity, CNS, hind limb unloading, microglia, ROS, MCAT

## Abstract

Microgravity (modeled by head-tilt bedrest and hind-limb unloading), experienced during prolonged spaceflight, results in neurological consequences, central nervous system (CNS) dysfunction, and potentially impairment during the performance of critical tasks. Similar pathologies are observed in bedrest, sedentary lifestyle, and muscle disuse on Earth. In our previous study, we saw that head-tilt bedrest together with social isolation upregulated the milieu of pro-inflammatory cytokines in the hippocampus and plasma. These changes were mitigated in a MCAT mouse model overexpressing human catalase in the mitochondria, pointing out the importance of ROS signaling in this stress response. Here, we used a head-tilt model in socially housed mice to tease out the effects of head-tilt bedrest without isolation. In order to find the underlying molecular mechanisms that provoked the cytokine response, we measured CD68, an indicator of microglial activation in the hippocampus, as well as changes in normal in-cage behavior. We hypothesized that hindlimb unloading (HU) will elicit microglial hippocampal activations, which will be mitigated in the MCAT ROS-quenching mice model. Indeed, we saw an elevation of the activated microglia CD68 marker following HU in the hippocampus, and this pathology was mitigated in MCAT mice. Additionally, we identified cytokines in the hippocampus, which had significant positive correlations with CD68 and negative correlations with exploratory behaviors, indicating a link between neuroinflammation and behavioral consequences. Unveiling a correlation between molecular and behavioral changes could reveal a biomarker indicative of these responses and could also result in a potential target for the treatment and prevention of cognitive changes following long space missions and/or muscle disuse on Earth.

## 1. Introduction

Bedrest, sedentary lifestyle, and isolation have negative effects on almost all systems of the human body, and understanding these effects has become a primary objective for enabling long-term space flights. Exposure to microgravity is associated with skeletal muscle atrophy, but it might also have detrimental effects on the central nervous system (CNS) and cognition, since spaceflight results in significant volumetric changes in brain grey matter [1]. Furthermore, simulated microgravity in mice induced impairments in immune functions [2]. Microglia, the resident immune cells of the CNS, are crucial for maintaining a healthy neural environment promoting homeostasis and responding to infection, or injury. Microglial activation can occur in response to a variety of triggers and is dependent on the type of pathological stressor or event [3]. Once activated, microglia not only primarily act as scavenger cells, removing debris and dying neurons [4], but also perform tissue repair and neural regeneration [5]. Microglia also act as second messengers by releasing a number of substances such as cytokines, chemokines, growth factors, and reactive oxygen species (ROS) [4,6]. Prolonged activations can lead to neuronal damage or death, as well as the activation of astrocytes, resulting in further endangerment to neurons and the further recruitment of microglia, thereby perpetuating the inflammatory state [7,8]. There are a number of potentially useful markers for specific microglia states [9]. Cluster of Differentiation 68 (CD68) is a lysosomal protein expressed at low levels by resting microglia and at high levels by activated phagocytic microglia [7,10,11]. CD68 is commonly used to visualize activated microglia in inflammatory responses in the brain and is potentially a suitable candidate for further exploring microgravity-induced microglial activation.

Studies have shown that microgravity and bedrest stimulate changes in immune cell response. Monocytes isolated from astronauts exhibit impaired function (reviewed in [12]). Furthermore, plasma concentrations of TNFα, IL-8, IL-1a, growth factors, and leukocyte recruitment chemokines have been observed to be increased in plasma following spaceflights [13]. Following the stimulation of lipopolysaccharides (LPSs), the monocyte secretion of IL-6, TNFα, and IL-10 significantly reduced, while IL-1β was elevated postflight [14]. During spaceflight, increased white blood cell count and decreased T cell function have been observed in astronaut crews [15]. Studies using blood collected from human bedrest models of head-down tilt-simulating headward fluid shifts in flight found changes in immune cells [16] and cytokine levels. After two-week hindlimb unloading (HU), a rodent model commonly used to simulate weightlessness, microgravity-induced cephalic fluid shifts, bedrest and anhedonia, the mRNA expression of numerous genes impacting immune response, synaptic plasticity, learning, and memory was altered in mouse brains [17]. HU also increased microglia activations in spinal cord [18] and neural stem cells [19]. We previously observed an increase in IL-3, IL-1β, IL-10, and IL-17 in the hippocampus after prolonged HU [20], cytokines that are known to be produced and secreted by activated microglia [8,21], and astrocytes [22,23]. Altered gravity was also observed to downregulate ICAM-1 expression in vitro in murine BV-2 microglial cells, while a subtype of smaller cells of BV-2 microglial cells appeared and was assumed to be activated cells [24]. Overall, previous studies showed that microgravity and bedrest both induced immune response in the CNS, but the microglial activations in the hippocampus in in vivo mouse models have not yet been investigated. We hypothesized that head-tilt bedrest will result in microglial activations in the hippocampus.

It has been established that spaceflight and spaceflight-like stressors cause oxidative stress responses within the hippocampus [25]. Due to the incomplete mitochondrial metabolization of oxygen, superoxide, hydrogen peroxide, and hydroxyl are generated. Behavioral and cognitive changes were associated with altered measures of oxidative stress, lipid peroxidation, and protein and DNA oxidation in the amygdala and hippocampus, brain regions implicated in anxiety and spatial memory [26]. The increased production of mitochondrial reactive oxygen species (ROS) leads to increased cytokine release from microglial cells, potentially indicating increased microglial activations [27,28]. Mitochondrial ROS is generated by activated microglia [29,30], and reducing its production is believed to be a suitable target to prevent the over-activation of microglia [27,28,31]. A mouse model for mitochondrial ROS quenching via the overexpression of the human catalase gene targeted toward the mitochondria (MCAT) has been previously utilized to investigate oxidative stress. MCAT mice have increased lifespan [32], improved age-dependent pathologies [33], reduced radiation-induced neurogenesis depletion [34], cognitive dysfunction, improved outcomes in models of neurological conditions including ataxia-telangiectasia [35], ALS [36], and Alzheimer’s disease [37]. MCAT mice also exhibit improved hippocampal spatial learning and contextual fear memory [26]. Previously, we showed that MCAT mice were protected from simulated microgravity-induced increases in cytokine expression in hippocampus. In our lab, in the MCAT mice, ROS quenching was demonstrated by measuring 4-HNE [38,39], catalase activity, and lipid peroxidation. We have also seen a strong genotype effect in the MCAT mice, with a significantly lower protein abundance of pro-inflammatory cytokines in the hippocampus. In a screen of forty-four cytokines and chemokines, fourteen (IFNγ, IL-4, IL-6,IL-7, IL-9, IL-10, IL-17, MCP-1, MCSF, RANTES, MCP-5, and IP-10) were downregulated in MCAT vs. WT mice [20]. These results suggest that the increased expression of these cytokines in wildtype hindlimb-unloaded mice is indictive of microglial activations and that mitochondrial ROS play an important role in microglial activation; thus, in this study, we used the MCAT mouse model to see if ROS quenching will mitigate HU-induced microglial activation.

Understanding the correlations between molecular changes in the brain, behavior, and plasma markers could refine our understanding of the kinetics of neurodegenerative changes and help find early biomarkers before irreversible damage occurs. Increased cytokine expression and neuroinflammation are associated with changes in behavior. There is evidence that inflammatory factors TNFα and IL-1β are associated with increased sick behaviors [40,41,42]. Theses cytokines have also been implicated in anorexic-like behavior [43]. IFNα is known to induce depression in humans [44], and TNFα is involved in depressive-like behavior as well [45]. In a mouse model, HU resulted in anxiety-like behaviors, which were associated with significant changes in the pattern of neural oscillations in the hippocampus [46]. Oxidative stress enhanced this response, and the inhibition of ROS reduced HU-induced anxiety [47].

We previously reported significantly reduced CD4+ T cell numbers and elevation in twelve cytokines in singly, but not socially, housed mice under normal loading conditions [48]. Singly housed mice exposed to simulated microgravity downregulated only one cytokine, IL-13, which is involved in anti-inflammatory mechanisms, and these changes were mitigated in the MCAT mouse model [20]. These data suggest that, under these conditions, social isolation and HU may reflect an epistatic interaction. In this study, we investigated the role of HU alone, without the additional isolation effect, in socially housed animals. MCAT mice were utilized to explore the association between oxidative stress and microglia activation in simulated microgravity. Correlations between microglia activation, cytokine expression and behavior were calculated to further our understanding of the effects of altered gravity on the CNS. In this study, we aim to determine the role of microglia, ROS pathways, and behavior, following head-tilt bedrest to elucidate the underlying molecular mechanism behind neuroimmune changes, thus paving the way for future therapies. We hypothesize that head-tilt bedrest will affect microglia activation in the hippocampus and that, in the MCAT model, quenching ROS will mitigate these changes.

## 2. Materials and Methods

### 2.1. Animals and Strains

In this study, a subset of the socially housed (2 per cage) sixteen-week-old female wild type C57BL/6NJ and MCAT transgenic mice was utilized as described in [20] and [48]. Wild type (WT) and MCAT mice were generated at NASA Ames Research Center (ARC) by crossing male MCAT mice, B6. Cg-Tg (CAG-OTC/CAT) 4033 Prab/J with female C57BL/6NJ mice (from Jackson Laboratory, Bar Harbor, ME, USA). Genotyping was performed using the Red’N Amp PCR kit following the manufacturer’s protocol (Millipore-Sigma, The Woodlands, TX, USA). All animal procedures were performed in compliance with protocols approved by the Institutional Animal Care and Use Committee (IACUC NAS-16-007-Y2) at NASA Ames Research Center (ARC).

### 2.2. Hindlimb Unloading to Produce Head-Down Tilt

Mice were assigned to one of four experimental groups (See Supplementary Figure S1; n = 8/group unless otherwise stated) and housed in a custom caging system as previously described [48]. In accordance with our previous work and with most space rodent studies to date, we chose females for this study. Socially housed, normally loaded (NL) controls were maintained in standard vivarium cages. All HU groups were acclimated for three days in HU cages prior to the onset of HU. Room temperature was maintained at a range of 23–24 °C with a 12 h light: dark cycle (6 AM:6 PM lights on:off). Food and water were provided ad libitum. Nestlets (Ancare, NY, USA) were provided for enrichment. Animal weights and food consumption were monitored throughout the duration of the experiment. Animals were euthanized 30 days after the start of HU via CO_2_ inhalation followed by cervical dislocation.

### 2.3. Sample Collection and Immunohistochemistry

The left-brain hemisphere was flash frozen and a tissue biopsy (1.20 mm Harris micro punch) from the hippocampus was performed inside a cryotome chamber set at −20 °C to avoid thawing. Hippocampal tissue was then homogenized in a mild lysis buffer (Tris 50 mM, NaCl 150 mM, Igepal 1%, Protease Inhibitors (Millipore-Sigma, The Woodlands, TX, USA) [49]. Samples were then centrifuged at 4 °C at 1000× *g* for 10 min and supernatants were aliquoted and flash frozen at −80 °C until analysis.

Cytokine protein abundance in hippocampal homogenates was analyzed using a Mouse Cytokine/Chemokine Array 44-Plex-MD44 (EVE Technologies, Calgary, AB, Canada) in which concentration standards are run for each cytokine. To verify the Multiplex method, IL-3 cytokine expression was validated by ELISA (Abcam, Cambridge, UK, Cat# ab113345-IL-3). Hippocampal cytokine levels were normalized to total protein content as determined by a BCA assay (Thermo Fisher, Waltham, MA, USA, Cat# 23225). Blood was collected from the vena cava immediately after euthanasia. Undiluted plasma was analyzed for cytokine protein abundance as described for hippocampal homogenates. The results and raw data of plasma and hippocampal cytokines are shown in Rubinstein et al., 2021 [20], and used here for correlations.

The right brain hemisphere was embedded in 4% PFA overnight and transferred the next day into 30% sucrose-PBS solution for cryoprotection. After sucrose impregnation, brains were embedded in OCT (OCT compound 4583, Sakura, Nagano, Japan) and frozen at −80 °C until cryosectioning. The brains were sectioned in a cryostat at 40 microns and stained for CD68. Slide-mounted sections were 1x-TBS-buffered at a pH of 7.3 and washed in H_2_O_2_ for antigen unmasking. Next, the primary antibody, rat-αmouse CD68 (Abcam ab53444, dilution 1:2000), was introduced in blocking-buffered 3% Normal Rabbit Serum After additional TBS washes, the secondary antibody was introduced (biotinylated rabbit-αrat, dilution 1:200 (Vector BA4001) in blocking-buffered 3% Normal Rabbit Serum). Amplification and fluorescent staining were performed with a Fluorescein Tyramide System (Perkin Elmer, Boston, MA, USA, NEL701A001KT), as described by the manufacturer. The indiscriminate nuclear staining agent 4′,6-diamidino-2-phenylindole (DAPI, Vector Labs, Mowry Ave Newark, CA, USA Vectashield, H-1200-10) was used to identify hippocampal regions, and the Allen Brain Atlas was used for reference (http://mouse.brain-map.org/) We chose to analyze 4 brains from each group except NL MCAT group in which 5 brains were analyzed, all with representative sections across the entire hippocampus (CA1/2, CA3 and dentate gyrus (DG)). We used 20X magnification, and 9–15 photos per hippocampus were taken. The unbiased microglia counting in each photo was performed manually counting microglia in each frame, using Image J program (https://imagej.nih.gov/ij/).

### 2.4. Mouse Behavioral Analysis

Video surveillance: A commercially available surveillance system that allows for both day and night image captures was utilized. The Lorex 4K UHD 8-channel Fusion NVR Security System with 8 “Smart Deterrence” 4K Cameras was utilized. This cage system was designed to not disturb ongoing behavior. The camera was placed horizontally to the cage, and it is affixed to a surface at a distance enabling visibility and the capture of full ambulatory motion and all species’ typical behaviors.

Video Sampling: Mice were recorded in their home cage continuously for a 24 h period, and samples of 15 min videos were analyzed using Excel to manually score the duration of various behaviors; time samples were at least 2 h away from the light-to-dark and dark-to-light transitions to ensure the acquisition of stable activity levels during each phase of the diurnal cycle.

We sampled 15 min from the following time points: 8 AM, 11:30 AM, 4 PM, 10 PM, 12 AM, 2 AM, and 4 AM. Each behavior graph represents the percentage of time the animal spent in engaging in a specific category from the total time measured.

Videos images were manually scored in Excel and analyzed for a range of typical mouse behaviors that would indicate normal functioning. To obtain a more general behavioral profile, behaviors were pooled into the following categories: “exploratory”, “social interaction”, “active”, and “rest/sleep”:

1. Exploratory Behavior: burrowing in bedding, climbing, exploratory sniffing, and exploratory ambulation;
2. Social Interaction: chasing, sniffing another mouse, allogrooming, nestlet social engagement, mounting, socializing with another mouse, and pulling another mouse.
3. Active Behavior: active behavior contains exploratory behavior, eating, drinking, sniffing, nestlet manipulation, self-grooming, ambulation, and all social interaction behaviors;
4. Rest/Sleep: rest/Sleep behavior contains sleep and non-directed movement.

### 2.5. Behavioral Categories Definitions

**Eating:** Mouth is on food. Mouse has visible food in its forepaws and is using its mouth to interact with food piece. **Drinking:** The animal rears up and licks the nozzle of the drinker. **Borrowing:** Burrowing in its bedding, which involves the paws and snout and exploring different cage parts. **Chasing:** Normally loaded animals only. Movement 3 or more steps towards the other mouse, while the other mouse is moving away. **Inactive:** Stationary in one spot with most of its body not moving. **Climbing:** Using paws to grip on cage in order to lift body and paws off the ground. **Sniffing other mouse:** One mouse’s nose in near proximity of another mouse. **Alogrooming:** Grooming that occurs when one mouse is grooming another mouse. **Non-directed movement (during rest/sleep):** Movement that is localized to a few grids (3 grids) and does not use more than three-fourths of the animal’s body. Sustained for no longer than 2 s. **Exploratory ambulation:** Movement that includes sniffing and meets the definition of Stanford’s four stages of exploration: search, attend, approach, and investigate while moving across multiple grids. **Nestlet social engagement** (described in Tahimic et al., 2019 [48]): In social HU, when one mouse extends beyond the halfway section of the shared cage and grabs a nestlet. **Nestlet manipulation:** Mouse directly touching nestlet with mouth and/or forepaws. **Mounting:** Normally loaded animals only. Mouse climbs on top of another mouse or has another mouse on top of it. **Sniffing (exploratory):** Moving ahead with the nose held in the air. **Ambulation:** Moving across multiple grids. **Self-grooming:** Mouse licking its own fur or grooming with paws. **With another mouse:** Proximity and interaction between mice. **Pulling another mouse:** While one mouse is climbing, the other pulls it by its mouth.

### 2.6. Statistical Analysis

Statistical analyses were performed using JMP (SAS), Version 14.0.0. Equal variances across groups were evaluated by Levene’s test. If variances were equal, a one-way ANOVA with Tukey’ or *t*-test post hoc was performed. For datasets with unequal variance and/or non-normal distributions, a non-parametric Wilcoxon all pairs test was performed with statistical significance set at *p* < 0.05. Correlations were performed in JMP (SAS), Version 14.0.0. via multivariate platform and non-parametric Spearman’s ρ test. For all analyses, the threshold for statistical significance was set at *p* < 0.05.

## 3. Results

### 3.1. HU Increased Activated Microglia in the Hippocampus

**Figure 1:**
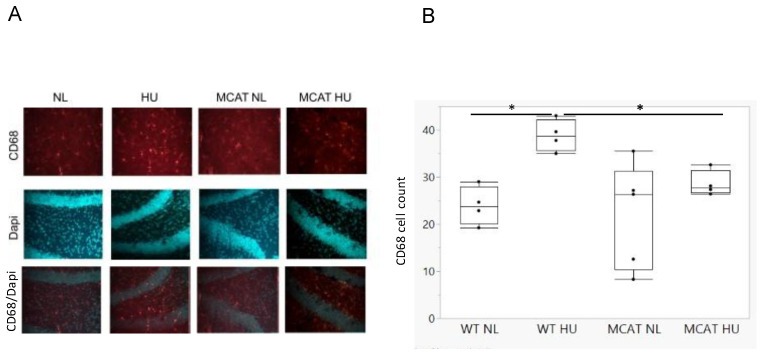
Hippocampus sections were stained, and CD6—expressing microglia were counted to quantify CD68 protein expression. (**A**). Sample of CD68 and DaPi staining in hippocampal sections, DG area). (**B**). CD68 expressing microglia counts, mean ± SE. * Statistically significant at *p* < 0.05 using a nonparametric Wilcoxon each pair comparison. Sample sizes: WT NL = 4; WT HU = 4; MCAT NL = 5; and MCAT HU = 4.

Increased CD68 levels are indicative of increased microglial activation. Hippocampal sections were stained to visualize and quantify CD68 protein expression (Figure 1A). Wild-type HU mice had a significantly greater number of CD68+ cells than normally loaded wild-type mice (Figure 1B). Interestingly, MCAT HU mice had a significantly lower CD68 count than wild-type HU mice (Figure 1B), indicating that mitochondrial catalase expressions can reduce HU induced microglia activations in the hippocampus.

### 3.2. Correlation of CD68 Count and Hippocampal Cytokines

**Figure 2:**
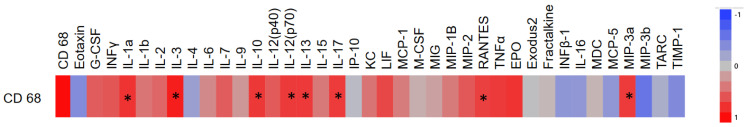
Correlation analysis between hippocampal CD68 count and changes in protein expression of hippocampal cytokines. Red color indicates positive correlations, and blue color indicates negative correlations. * Statistically significant at *p* < 0.05 using nonparametric Spearman’s.

Correlation analysis was performed between hippocampal CD68 count and protein expression of hippocampal cytokines (cytokine raw data are described in [20,50]). In total, the expression of eight proteins (IL-1a, IL-3, IL-10, IL-12, IL-13, IL-17, RANTES, and MIP-3a) had a significant positive correlation with hippocampal CD68 counts (Figure 2). Five pro-inflammatory cytokines were previously identified to be affected by [20], and three of these cytokines are among the ones shown here to have a significant correlation between CD68 counts and cytokine profiles in the hippocampus (IL-3, IL-12, and IL-17).

### 3.3. Changes in Behavior Observed during Light and Dark Cycles

**Figure 3:**
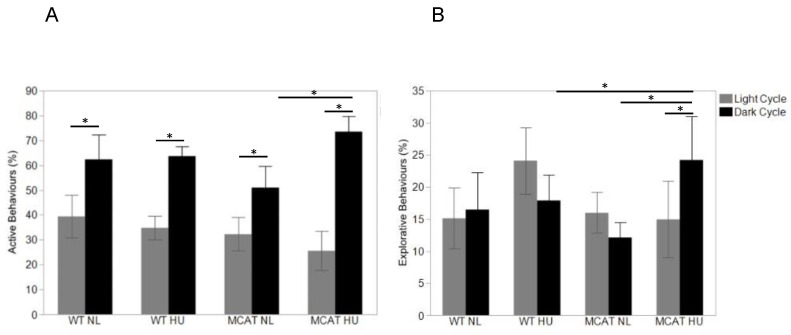
Observed animal behaviors during day (light) and night (dark) cycle. (**A**). Categorized into “active” behavior expressed as the percentage of time from the total time measured, mean ± SE. Sample sizes: WT NL Light = 7; WT NL Dark = 7; WT HU Light = 8; WT HU Dark = 8; MCAT NL Light = 8; MCAT NL Dark = 8; MCAT HU Light = 7 and MCAT HU Dark = 8. * Statistically significant at *p* < 0.05, using one-way ANOVA followed by *t*-test comparing each pair post hoc. (**B**) Categorized into “Exploratory” behavior expressed as percent of time from total time measured, mean ± SE. Sample sizes: WT NL Light = 7; WT NL Dark = 7; WT HU Light = 8; WT HU Dark = 8; MCAT NL Light = 8; MCAT NL Dark = 8; MCAT HU Light = 7 and MCAT HU Dark = 8. * Statistically significant at *p* < 0.05 using nonparametric Wilcoxon.

**Figure 4:**
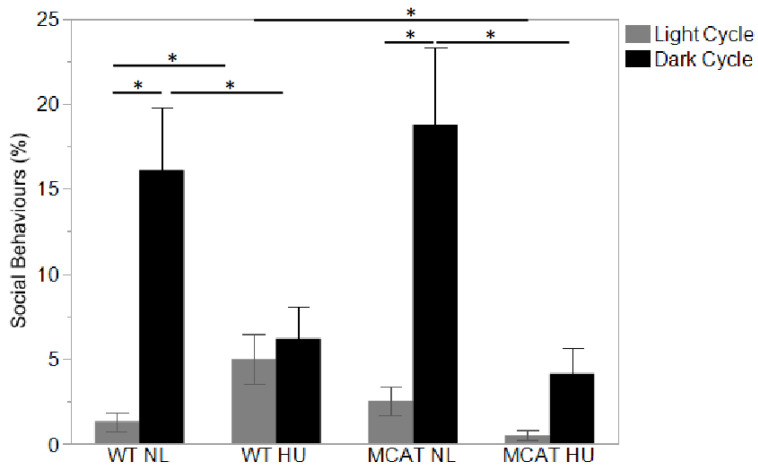
Observed animal behaviors during day (light) and night (dark) cycle categorized into “social interaction”. Behavior expressed as the percentage of time from the total time measured, mean ± SE. Sample sizes: WT NL Light = 7; WT NL Dark = 7; WT HU Light = 8; WT HU Dark = 8; MCAT NL Light = 8; MCAT NL Dark = 7; MCAT HU Light = 7 and MCAT HU Dark = 8. * Statistically significant at *p* < 0.05 using nonparametric Wilcoxon.

**Figure 5:**
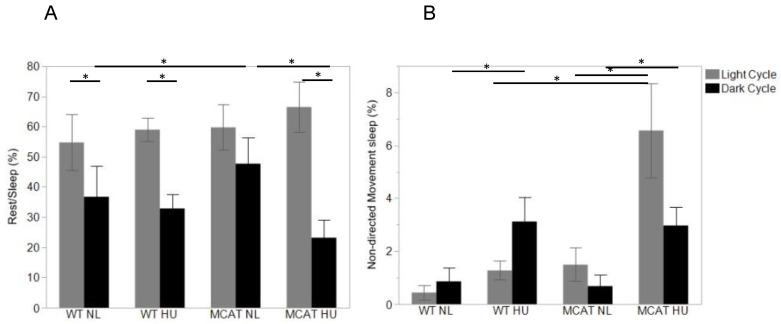
Observed animal behaviors during day (light) and night (dark) cycles. (**A**) Categorized into “rest/sleep”. Behavior expressed as the percentage of time from the total time measured, mean ± SE. Sample sizes: WT NL Light = 7; WT NL Dark = 7; WT HU Light = 8; WT HU Dark = 8; MCAT NL Light = 8; MCAT NL Dark = 7; MCAT HU Light = 7 and MCAT HU Dark = 8. * Statistically significant at *p* < 0.05, using one-way ANOVA followed by *t*-test comparing each pair post hoc. (**B**) Categorized into “non-directional movement during sleep”. Behavior expressed as the percentage of time from the total time measured, mean ± SE. Sample sizes: WT NL Light = 7; WT NL Dark= 8; WT HU Light = 7; WT HU Dark = 7; MCAT NL Light = 8; MCAT NL Dark = 8; MCAT HU Light = 7 and MCAT HU Dark = 8. * Statistically significant at *p* < 0.05 using nonparametric Wilcoxon.

Observed animal behaviors were categorized into “active” (Figure 3A), “exploration” (Figure 3B), “social interaction” (Figure 4), and “inactive” (including rest/sleep and non-directional movement during sleep) (Figure 5). During the light cycle, MCAT HU mice showed changes in behavior compared to wild-type HU mice, where reduced social behavior (Figure 4) and an increase in non-directed movement during sleep (Figure 5B) were observed.

Compared to light cycles, many more variations in behavior between most groups were observed during dark cycles. All groups showed increased active behavior compared to the light cycle, as expected. Only MCAT HU mice showed increased exploratory behavior during dark cycles compared to light cycles (Figure 3B). For social behavior, only normally loaded mice showed increased behavior during dark cycles compared to light cycles (Figure 4). All groups but normally loaded MCAT mice had decreased rest/sleep behavior during dark cycles compared to light cycles (Figure 5A).

During the dark cycle, MCAT HU mice showed higher exploratory behaviors compared to wild-type HU mice (Figure 3B) and higher active and exploratory behaviors compared to normally loaded MCAT mice (Figure 3A,B). For social behaviors during the dark cycle, wild-type HU mice showed decreased behavior compared to normally loaded wild-type mice (Figure 4), while MCAT HU mice also showed decreased behavior compared to normally loaded MCAT mice (Figure 4). For inactive behavior, rest/sleep activities of normally loaded MCAT mice were greater than normally loaded wild-type mice, where MCAT HU mice were lower than normally loaded MCAT mice (Figure 5A). Moreover, for non-directional movements during rest, wild-type HU mice had more activities than normally loaded wild-type mice, and MCAT HU mice had more activities than normally loaded MCAT mice (Figure 5B).

### 3.4. Correlations of Categorized Behaviors with Plasma and Hippocampal Cytokines

**Figure 6:**
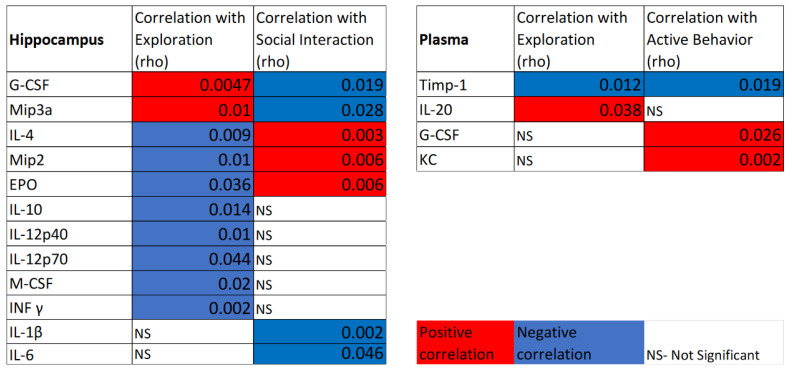
Correlations between categorized animal behavior during dark cycles (in which animals perform most activity) and cytokine protein expression in the hippocampus and plasma. Red color indicates positive correlation and blue color indicates a negative correlation; the numbers represent the *p*-value of the probability of multivariate pair-ways nonparametric Spearman’s correlation (JMP-SAS). NS—nonsignificant. Statistical significance determined at *p* < 0.05 using nonparametric Spearman’s.

Next, we assessed correlations between categorized animal behavior during dark cycles (in which animals perform most activities) and cytokine protein expression in the hippocampus and in plasma (Figure 6) with the aim to identify potential biomarkers of simulated microgravity consequences. In the hippocampus, correlations, either positive or negative, were observed in 12 cytokines. The activity category with the most correlations was explorative behavior, which was correlated positively with G-CSF and Mip3a and negatively with INF γ, IL-4, IL-10, IL-12p40, IL-12p70, M-CSF, Mip2, and EPO (Figure 6). Next, social interactions were positively correlated with IL-4, Mip2, and EPO and negatively with G-CSF, IL-1β, IL-6, and Mip3a (Figure 6). Inactive behavior correlated positively with INF γ (rho- 0.03, data not shown). For plasma cytokines, active behavior was positively correlated with G-CSF and KC and negatively correlated with Timp1. Inactive behavior was negatively correlated with KC and positively with Timp1 (rho- 0.003 and 0.02, respectively, data not shown). Exploration was positively correlated with IL-20 and negatively with Timp1 (Figure 6).

## 4. Discussion

Microgravity experienced during prolonged spaceflight results in neurological consequences, CNS dysfunction, and potentially impairment in performances [51]. Rodent hindlimb-unloading head-down tilt models have been utilized in ground-based studies to elucidate stress responses in numerous tissues, the immune system, and CNS [52]. Further investigations on the mechanisms of these responses are paramount for the identification of potential biomarkers as well as the development of future countermeasures. Previously, we found that isolation also may impact the response to microgravity by masking its effects [20]. Thus, in this current study, we were interested in assessing the effect of simulated microgravity (without isolation with mice socially housed) on a subset of immune responses of the hippocampus and behavioral outcomes.

The hippocampus is crucial for learning and memory, and any alterations in this region can have detrimental consequences on CNS function. HU in mice led to protein changes in the hippocampus, primarily in structural proteins and proteins involved in metabolism [53]. A previous study reported the upregulation of CD68 in the soleus [54] and increased microglia in spinal cord of male mice [18] that underwent HU. However, there remains a gap with respect to HU-induced CD68 responses in the brain. We found, in female mice that underwent HU, increased CD68 in the hippocampus, indicating an HU-induced increased activation of microglia. Deep-space missions will expose crew members to higher levels of radiation in addition to exposure to altered gravity. The exposure of low doses of high-LET radiation can induce inflammatory processes and can change cognition and behaviors [55]. Additionally, sex differences in cortical levels of CD68 and BDNF were observed in radiation exposure [56]. In accordance with our previous work and with most relevant space rodent studies to date [55,57,58], we chose females for this study. The additional reason for using females in our study was that, in studying male behavior, there are a lot of masking effects due to male-to-male aggression when housed in the same cage, and here, social housing was utilized. With the growing number of female astronauts, identifying these responses in females to space environment stressors is increasingly important. Sex differences are an important factor in the hippocampal stress response; thus, we plan to explore these responses to simulated spaceflight in our future studies and to assess if similar results will be seen in males.

There are several limitations of the HU model vs. spaceflight. During HU, the forelimbs, head, and upper back remain weightbearing vs. the entire body unloading in space. The HU model does not include the stress of launch and landing, and fluid shifts are not identical. Despite these limitations, this model is widely used and is instrumental in demonstrating physiological changes that occur during unloading [59].

Sleep and ROS quenching have a reciprocal relationship; when there is a lack of sleep, the accumulation of ROS might cause adverse effects [60]. Melatonin, a sleep-inducing agent, decreased abnormal protein nitration, the accumulation of Aβ polypeptides, and extended the lifespan in mice Alzheimer’s model brains [61]. In our study, as expected, mice rest less during the dark cycle; interestingly, in the WT MCAT group, mice were sleeping for the same period of time during the day and night, which could indicate a shift in their circadian rhythm. HU mice present more non-directed movements during rest compared to WT, which is probably led by the discomfort of HU, and these differences will be investigated further in our future studies.

Chronic stress is a risk factor for various mental and degenerative disorders, both the hypothalamic–pituitary–adrenal axis and the sympathetic nervous system are activated and elicit glucocorticoids and noradrenaline, respectively. These stress pathways have both simulating and suppressant effects on microglia depending on the specific environment [62]. Cytokines not only target microglia [8] but are also secreted by them, and their interactions differ in normal (where cytokines work as signaling molecules) vs. pathological states, where these pathways may spin out of control (e.g., cytokine storm) and cause a cycle of chronic neuroinflammation. Acute and chronic stress, a known circumstance of prolonged spaceflight, have also been shown to induce microglia activation resulting in an enhanced release of pro-inflammatory cytokines [10]. Mice that undergo HU have increased circulatory levels of corticosterone [20,48], an indicator of stress. We previously observed that HU resulted in changes in the expression of five hippocampal cytokines. Four out of these five cytokines (IL-3, IL-1β, IL-10, and IL-17) were upregulated in HU versus NL mice, while IL-12 was downregulated. Additionally, HU elevated neutrophils and the neutrophil-to-lymphocyte ratio [63] and IL-20 in plasma [20]. Here, hippocampal CD68 was correlated with eight cytokines and chemokines, primarily those involved with T cell responses, further indicating disruptions in the inflammatory status. Prolonged neuroinflammation is associated with cognitive deficits, mood disorders, and stress-related behavior [64]. In this study, twelve hippocampal cytokines and five circulatory cytokines were correlated with changes in exploratory and social behavior during the dark cycle. Additionally, we saw that hippocampal IL-1, IL-13, and Mip3a have positive correlations with both hippocampal microglia activation and negative correlations with social behavior. Similarly, hippocampal IL-10 and IL-12p70, which are positively correlated with activated microglia, have negative correlations with explorative behavior. IL-1 and IL-6 have previously been linked to sick behavior and fatigue, [65] and when neutralized, they ameliorated specific neuropsychiatric symptoms [66]. IL-10 and IL-12 are both produced by macrophages and dendritic cells. IL-12 is strongly implicated in the pathogenesis of inflammatory neurological diseases, such as multiple sclerosis. Mip-3a is a chemokine recently shown to have an important role in the autoimmune pathogenesis of the central nervous system. IL-13 has been shown to control brain inflammation by enhancing activated microglia death and enhancing neuronal survival [67].

It has been suggested before that there is an interplay between cytokines in the CNS and behavior, which probably evolved to promote healing and self-isolating behavior to protect other individuals from becoming infected. However, when this balance is disturbed in age-related cognitive impairments and/or mood disorders, similar immune changes are observed: for example, elevated CCL-11 plasma levels in schizophrenic patients [68]. The negative correlation of the cytokines found here with exploratory behavior, together with the elevated microglial state in the hippocampus, might illuminate similar pathways. It has been shown that IL-10 and IL-12 (P70) levels predict the risk of COVID-19 progression in hypertensive patients [69]; in s similar manner, these cytokines could be used as biomarkers for behavioral and molecular changes driven by neuroinflammation. It is still unclear if cytokines drive stress-induced sickness behaviors or are a consequence of it, but since the impact of these changes is crucial to many behavioral pathologies, finding more links between immune and behavior changes is important for developing more specific therapies.

Altered expression levels in the plasma that correlate with inactive behavior (Timp1 and KC) could be useful as biomarkers for astronauts or patients with sedentary lifestyle. The limitation of the correlations found in this study lays in its limited sample number; thus, these results must be verified in larger groups. Previous studies have shown that hindlimb unloading affects the blood–brain barrier’s integrity [70]; in future studies, it would be also interesting to assess whether peripherally infiltrating microglia such as monocytes are present in the brain of hindlimb-unloaded animals.

**Figure 7:**
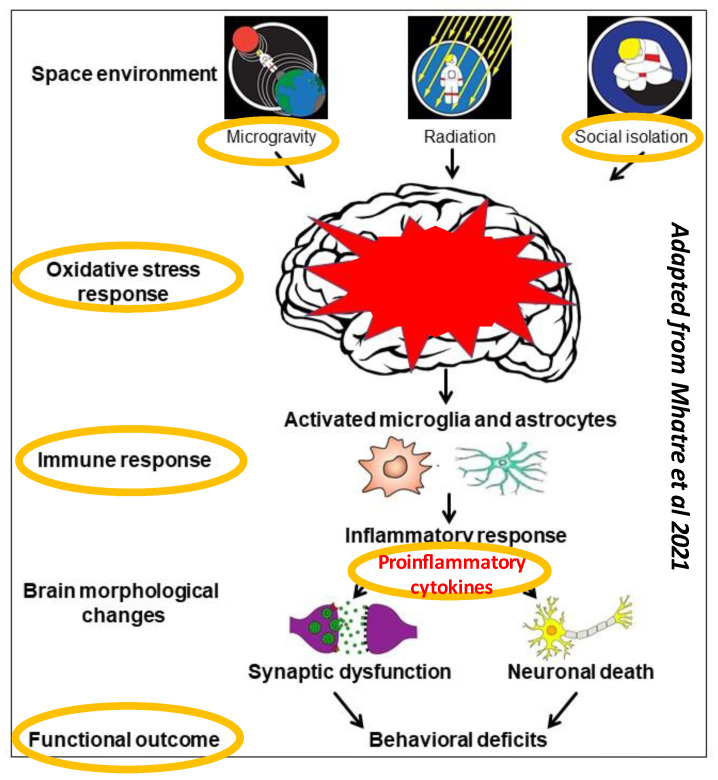
A schematic cascade of the neuro-consequences of spaceflight adapted from Mhatre et al. [48] with our findings highlighted (in orange oval).

A prolonged inflammatory state has been linked to an increase in oxidative damage [71,72]. Spaceflight and ground-based models have demonstrated oxidative stress affecting the brain and immune response, as reviewed previously [51,71]. Additionally, oxidative stress is associated with physiological and cellular changes in the CNS [71,73]. In an animal chronic social defeat, observed deficits in behavior were attributed to elevated levels of ROS and activated microglia. While activated microglia release pro-inflammatory cytokines, microglia are also activated by cytokines released by damaged neurons. HU in mice has been shown to induce oxidative stress in the brain, resulting in lipid peroxidation and the activation of NF-κb [25], a transcription factor that induces the expression pro-inflammatory cytokines. This in turn, induces the formation of ROS, resulting in increased pro-inflammatory cytokines and microglia activation further perpetuating the cycle and a chronic inflammatory state. Therefore, we tested whether mitochondrial ROS is a potential mechanism for neuroimmune deficits caused by simulated microgravity. Previously we saw that mitochondrial ROS quenching reduced cytokine protein levels in the hippocampus and mitigated the effects of HU and of social isolation [20]. Here, we observed a reduction in hippocampal CD68 in MCAT mice that underwent HU compared to the WT, indicating an important role of ROS in HU-induced microglia activation and that it is a potential target for countermeasures (Figure 7).

Unveiling a correlation between molecular and behavioral changes could reveal a biomarker indicative of these responses and could also result in a potential target for the treatment/prevention of cognitive changes astronauts experience due to prolonged spaceflight. Microglia activation may be suitable candidates for examining this relationship. Microglia have been implicated in a number of neurological disorders, including multiple sclerosis, Alzheimer’s disease, strokes, amyotrophic lateral sclerosis, and Parkinson’s disease [3], and they may be appropriate targets for prevention or treatment. Overall, our study sheds new light on the underlying mechanism of how simulated microgravity causes neuroinflammation, implicating microglial brain activation, and links this process to behavioral consequences. Additionally, we show that microglial activation in the hippocampus caused by simulated microgravity is mitigated by ROS quenching. Hence, in future studies, it would be interesting to investigate antioxidant treatments in both astronauts and patients with sedentary lifestyles.

## Supplementary Materials

The following supporting information can be downloaded at: https://www.mdpi.com/article/10.3390/life12111838/s1, Figure S1: Experimental design.
