# Supplementary material for: Mitochondria-Targeted Human Catalase in the Mouse Longevity MCAT Model Mitigates Head-Tilt Bedrest-Induced Neuro-Inflammation in the Hippocampus
Source: Life (Basel). 2022 Nov 9;12(11):1838. doi: 10.3390/life12111838 (PMC9695374; doi:10.3390/life12111838)
Supplement: Supplementary file 1 [file life-12-01838-s001.zip › life-1883406-supplementary.pdf]

Supplementary Materials for Mitochondria-Targeted Human Catalase in the Mouse Longevity MCAT Model Mitigates Head-Tilt Bedrest-Induced Neuro-Inflammation in the Hippocampus

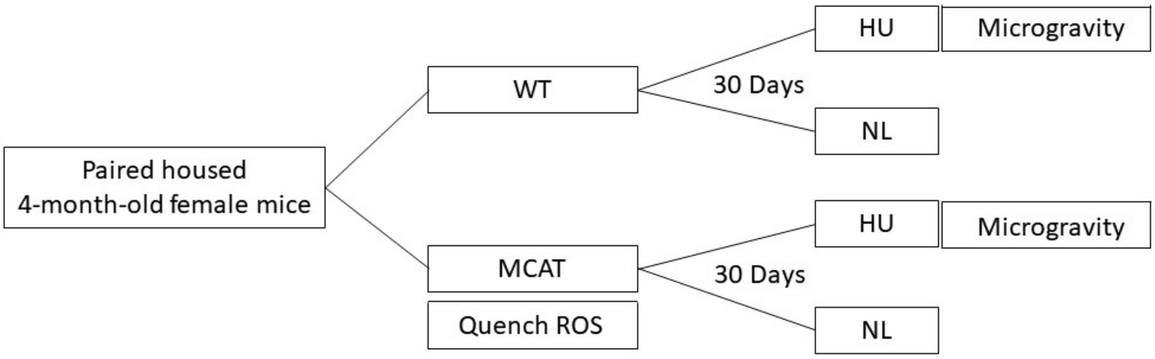

Figure S1. Experimental design: The mice used were a subset (only socially housed) wild type C57BL/6NJ and MCAT transgenic mice, and sixteen-week-old females were assigned to one of four experimental groups: WT (Wild Type) or MCAT (expressing human catalase in the mitochondria), NL (normally loaded), or HU (hind-limb unloaded); N = 8 per group unless otherwise stated and housed in pairs in a custom caging system, as previously described in [20,48].
